# Isolation and Mechanistic Characterization of a Novel Zearalenone-Degrading Enzyme

**DOI:** 10.3390/foods11182908

**Published:** 2022-09-19

**Authors:** Jian Ji, Jian Yu, Wei Xu, Yi Zheng, Yinzhi Zhang, Xiulan Sun

**Affiliations:** 1 State Key Laboratory of Food Science and Technology, School of Food Science and Technology of Jiangnan University, Wuxi 214122, China; 2 College of Food Science and Pharmacy, Xinjiang Agricultural University, Ürümqi 830052, China; 3 Key Laboratory for High-Tech Research and Development of Veterinary Biopharmaceuticals, Jiangsu Agri-Animal Husbandry Vocational College, Taizhou 225300, China

**Keywords:** zearalenone, enzyme, biodegradation, structural analysis

## Abstract

Zearalenone (ZEN) and its derivatives pose a serious threat to global food quality and animal health. The use of enzymes to degrade mycotoxins has become a popular method to counter this threat. In this study, *Aspergillus niger* ZEN-S-FS10 extracellular enzyme solution with ZEN-degrading effect was separated and purified to prepare the biological enzyme, FSZ, that can degrade ZEN. The degradation rate of FSZ to ZEN was 75–80% (pH = 7.0, 28 °C). FSZ can function in a temperature range of 28–38 °C and pH range of 2.0–7.0 and can also degrade ZEN derivatives (α-ZAL, β-ZOL, and ZAN). According to the enzyme kinetics fitting, ZEN has a high degradation rate. FSZ can degrade ZEN in real samples of corn flour. FSZ can be obtained stably and repeatedly from the original strain. One ZEN degradation product was isolated: FSZ−P(C_18_H26O_4_), with a relative molecular weight of 306.18 g/mol. Amino-acid-sequencing analysis revealed that FSZ is a novel enzyme (homology < 10%). According to the results of molecular docking, ZEN and ZAN can utilize their end-terminal carbonyl groups to bind FSZ residues PHE307, THR55, and GLU129 for a high-degradation rate. However, α-ZAL and β-ZOL instead contain hydroxyl groups that would prevent binding to GLU129; thus, the degradation rate is low for these derivatives.

## 1. Introduction

Zearalenone (ZEN) is a non-steroidal estrogen mycotoxin biosynthesized by several Fusarium species. It was first discovered from moldy corn in 1962 [1]. ZEN often pollutes food crops, such as corn and wheat, thereby affecting the health of humans or animals. Therefore, there is significant interest in the identification of effective methods to degrade ZEN for improved food security. ZEN degradation requires the destruction of the original structure of ZEN or the formation of low-toxic substances through biological metabolic processes [2]. Through these metabolic processes, ZEN will also produce a variety of derivatives, such as: α-Zearalanol(α-ZAL), Taleranol(β-ZOL), Zearalanone(ZAN), Zearalenone-14-sulfate (Z14S), and Zearalenone-14-glucoside (Z14G). Among these derivatives, Z14S and Z14G have lower estrogen toxicity [3,4].

Of the ZEN degradation methods, biodegradation is of particular interest due to its good degradation efficiency and high safety. Several biological enzymes have been identified that can degrade ZEN. Kakeya et al. [5] isolated the ZEN-degrading enzyme, ZHD101, from *Clonostachys rosea* IFO 7063. Another group obtained peroxidase POD that can be used for the degradation of ZEN in beer by separation and purification [6]. Ref. [2] cloned and expressed the ZEN-degrading enzyme, ZENG, with high-heat resistance. Zhou et al. [7] isolated RmZHD, an enzyme that can be used for ZEN degradation. Other enzymes derived from various fungi and bacteria have also been shown to have ZEN degradation, including ZHD607 [8], ZENC [9], zlhy-6 [10], and BCT [11]. Although enzymes, such as ZHD101, can be used for the degradation of ZEN, the source strains are not food grade; thus, the safety of using these strains for food applications is unknown. These enzymes are also only relatively weakly active for the removal of other ZEN derivatives produced in food processing. These factors limit the application of these enzymes in the food industry. Therefore, it is important to identify high-efficiency, ZEN-degrading enzymes derived from food-grade strains.

In previous studies, we characterized a food-grade strain of *Aspergillus niger* FS10 that can be used to degrade a variety of mycotoxins [12,13]. We improved the metabolic activity of this strain through ZEN stress to obtain the metabolically enhanced *Aspergillus niger* zearalenone-stressed-FS10 (ZEN-S-FS10) [14]. Previous studies showed that *Aspergillus niger* ZEN-S-FS10 degrades ZEN using an extracellular enzyme secreted by its hyphae into the fermentation broth [14]. In this study, we used an *ÄKTA* protein purification and separation system to obtain the extracellular protein FSZ with ZEN degradation activity then explored the optimal degradation conditions for this enzyme, and analyzed its main degradation products. The amino acid sequence of FSZ was analyzed by amino acid sequencing and compared with the sequences of other ZEN-degrading enzymes. This enzyme has low homology with other degrading enzymes, indicating that this is a novel ZEN-degrading enzyme. The FSZ protein structure was predicted, and its interaction with ZEN was modeled. Finally, we tested the purified enzyme using corn flour and found improved degradation.

## 2. Materials and Methods

### 2.1. Cultivation of ZEN-S-FS10 and Its Crude Enzyme Solution

*Aspergillus niger* ZEN-S-FS10, a non-toxigenic filamentous fungus that was originally isolated from fermented food was obtained from the China General Microbiological Culture Collection Center (CGMCC NO.20745). Fungal 18S rDNA sequencing confirmed the strain as *Aspergillus niger* with gene homology of 99%. The sequencing results are shown in Table S1. For cultivation, autoclaved potato dextrose broth (PDB) was inoculated with spores of *Aspergillus niger* ZEN-S-FS10 at a spore concentration of 10^6^ CFU/mL. The culture was transferred to a shaker and grown for 28 h at 28 °C and 180 rpm. A Buchner funnel was used to filter out the grown hyphae by vacuum filtration, and the extracellular fermentation broth was retained. The protein content in the fermentation broth was measured by BCA, and protein activity in the fermentation broth was measured according to GB/T23527-2009 [12].

### 2.2. Purification of FSZ by ÄKTA Protein Purifier and SDS-PAGE Gel Electrophoresis

The cell-free supernatant was applied to a Superdex™ 30Increase 10/300 GL size exclusion chromatography column using the *ÄKTA* purifier platform (GE Healthcare, Marlborough, MA, USA) according to the described method [15]. Briefly, the following operating parameters were applied: phosphate-buffered saline solution (PBS) was used as the mobile phase with an equilibrium volume of two column volumes (CV), elution at pH 7.4, an elution volume of 1.5 CV, and a flow rate of 1.0 mL/min. Samples of 20 mL were collected when UV absorption was detected at 280 nm.

A 10% SDS-PAGE gel electrophoresis kit was used for protein gel electrophoresis (Beyotime Biotechnology, Shanghai, China) and separation was performed according to the kit instructions. The gel was stained with Coomassie Brilliant Blue R250 and then decolorized with ultrapure water containing 20% acetic acid for 24 h, as described [16]. A total of 10 μL of sample was loaded in each lane.

### 2.3. Detection of Enzyme Concentration and ZEN Degradation Activity

Enzyme activity and concentration were measured as follows. After elution, 1 mL samples were mixed with ZEN standard products (purchased from Enzo Life Sciences, Inc., Beijing, China) to a concentration of 1.0 μg/mL. The mixtures were placed in a shaker at 28 °C and 180 rpm for 24 h. High-performance liquid chromatography (HPLC) was used to observe ZEN degradation, as described [14]. The peak area corresponding to ZEN after degradation was determined, and the standard curve method was used to calculate the concentration, c_1_, of ZEN. The degradation rate was then calculated using Equation (1). Three biological replicates of each sample were assayed in parallel (*n* = 3).

(1) $$Degradation\;rate\left(\%\right)=\frac{1.0-c_{1}}{1.0}\times 100\%$$

### 2.4. Determination of the Optimum Active Conditions for FSZ

#### 2.4.1. Determination of Temperature Effects

Purified FSZ was mixed with ZEN samples and incubated in a shaker at 18 °C, 28 °C, 38 °C, or 48 °C at 180 rpm. Samples were removed every 12 min over 60 min, and the degradation rate of ZEN was detected, as described above. Three biological replicates of each sample were assayed in parallel (*n* = 3).

#### 2.4.2. Determination of pH Effects

The pHs of the purified FSZ and ZEN mixtures were adjusted by the addition of glacial acetic acid and ammonia water to pH = 2.0, 3.0, 4.0, 5.0, 6.0, 7.0, 8.0, 9.0, 10.0, or 11.0 before shaking for 28 h at 28 °C and 180 rpm. Samples were removed every 12 min over 60 min, and the degradation rate of ZEN was detected, as described above. Three biological replicates of each sample were assayed in parallel (*n* = 3).

#### 2.4.3. Influence of Metal Ion

To test the influence of different metal ions, mixtures of FSZ and ZEN were combined with 0.1 mol/mL Na^+^, Ca^2+^, K^+^, Mg^2+^, Zn^2+^, Fe^3+^, Cd^2+^, Mn^2+^, or EDTA before adjustment to pH = 7.0. in each group of samples. Samples were removed every 12 min over 60 min, and the degradation rate of ZEN was detected, as described above. Three biological replicates of each sample were assayed in parallel (*n* = 3).

#### 2.4.4. Influence of Ionic Strength

To test the influence of ion strength, mixtures of FSZ and ZEN were adjusted to Na^+^ concentration gradients of 0.1, 5, 10, 20, 50, or 100 mol/mL. The pH of each sample was controlled at 7.0. Samples were removed every 12 min over 60 min, and the degradation rate of ZEN was detected, as described above. Three biological replicates of each sample were assayed in parallel (*n* = 3).

### 2.5. Determination of the Kinetic Parameters of ZEN Degradation by FSZ

FSZ at the concentration with the best enzyme activity (6.0 U/mg) was combined with different concentrations of ZEN, and the kinetic parameters were determined. ZEN was tested at concentrations of 0.1, 0.2, 0.5, 0.8, 1, 1.5, 2, 3, 4, 5, or 6 μg/mL. The degradation was measured, and the results were used to calculate the maximum reaction rate (*V_max_*) and Michaelis constant (*K_m_*) from the Lineweaver–Burk (LB plot). The plot of $\frac{1}{v_{0}}$ and $\frac{1}{S}$ (LB plot) yields a straight line that is characterized by a slope of $\frac{K_{m}}{V_{max}}$ with its y-intercept at $\frac{1}{V_{max}}\;$ and x-intercept at $\frac{1}{K_{m}}\;$ [17].

In these measurements, ZEN standard was added to 50 mL FSZ enzyme solution with phosphate-buffered solution (PBS) as the solvent at a ZEN concentration of 1 μg/mL. The mixtures were incubated with shaking for 28 h at 28 °C and 180 rpm, and 1 mL samples were removed every 4 h. HPLC detection was performed to measure the ZEN residual concentration, and then the degradation rate was calculated. Three biological replicates of each sample were assayed in parallel (*n* = 3).

### 2.6. Measurement of Ability of FSZ to Degrade ZEN Derivatives

The abilities of FSZ to degrade common ZEN derivatives, α-ZAL, β-ZOL, and ZAN, were measured using 1 mL of the purified FSZ enzyme and 1 μg/mL derivative standards (Enzo Life Sciences, Inc., Beijing, China). The mixtures were incubated with shaking for 28 h at 28 °C and 180 rpm and then subjected to the ultra-high performance liquid phase mass spectrometry method [18] to detect the residual amount of the derivative and calculate the degradation rate. Three biological replicates of each sample were assayed in parallel (*n* = 3).

### 2.7. Exploration of ZEN Degradation Products

To further demonstrate the degradation products of FSZ to ZEN, we briefly explored the products before and after ZEN degradation. Take 1.0 mL of ZEN standard with a concentration of 1.0 μg/mL as blank control (ZEN group). An amount of 1.0 mL of ZEN-degrading enzyme FSZ obtained by the separation and purification described above was used as a sample control (FSZ group). The ZEN standard was added to the 1.0 mL FSZ sample described above, and the ZEN concentration was controlled to be 1.0 μg/mL as the experimental group (degradation group). Then it was put it into a shaker at 28 °C and 180 rpm for 28 h. The degradation products were analyzed and identified by high-resolution liquid chromatography–mass spectrometry (HPLC-qTOF-MS).

The detection method was carried out by Ultra Performance LC combined with AB SICEX 5600 system. UPLC was performed on a Waters Acquity UPLC system equipped with a UV detector. The column parameters used were a Waters UPLC HSS T3 column (1.8 μm, 2.1 mm × 100 mm). The column conditions were as follows: column temperature of 35 °C; injection volume of 5 μL; and flow rate of 0.3 mL/min. The mobile phases are as follows: positive mode A is 100% H_2_O (0.1% formic acid); positive mode B is 100% acetonitrile (0.1% formic acid); negative ion mode A is 100% H_2_O (0.5 mol/L NH4F); negative ion mode B is 100% supernatant pure water; injection volume of 5 μL; and flow rate of 0.3 mL/min. Other parameters were: capillary voltage of 4.5 kV; cone voltage of 30 kV; ion source temperature of 120 °C, and desolvation temperature of 350 °C. Mass spectra were performed in the scan range of m/z = 50–1000. MSDIAL 4.24 and MSFINDER 3.46 software were used to analyze the mass spectrometry data and speculate the possible degradation products and their structural information [19].

### 2.8. Sequence Analysis and Structural Characterization of FSZ

The band corresponding to FSZ was cut out of a protein gel and subjected to enzymatic protein digestion, peptide desalting, and mass spectrometry detection (sequencing performed by Shanghai Luming Biotechnology Co., Ltd., Shanghai, China). The amino-acid-sequencing results were uploaded to NCBI, and the Blast function was used. The amino acid sequence was also uploaded to the Clustal website for sequence alignment with previously reported ZEN-degrading enzymes to assess potential homology. The amino acid sequence was also uploaded to the SWISS-Model website and BIOVIA Discovery Studio(Server+Client) 2019 v19.1 (BIOVIA, San Diego, CA, USA) for structural simulation and analysis of the three-dimensional structure of the protein. We simulated docking of FSZ with ZEN and its derivatives to model the interaction.

### 2.9. Evaluation of the Ability of FSZ to Degrade ZEN in Corn Flour

To test the ability of purified FSZ to degrade ZEN in a food product, degradation was assayed in corn flour. To do this, 1 g of commercially available corn flour (ZEN content of about 1.0 μg/g) was weighed and dissolved in 1mL of purified FSZ enzyme solution. The mixture was incubated in a 28 °C, 180 rpm shaker and allowed to degrade for 28 h. The residual ZEN content was detected by high-performance liquid chromatography, as described above, and the relative degradation rate was calculated. Three biological replicates of each sample were assayed in parallel (*n* = 3). Rstudio (Rstudio, Boston, MA, USA) used for linear fitting was performed to determine the rigor of the detection method, and the relevant parameters were calculated.

## 3. Results and Discussion

### 3.1. ÄKTA Protein Purification of FSZ

#### 3.1.1. Purification of FSZ from Crude Enzyme Solution

**Figure 1:**
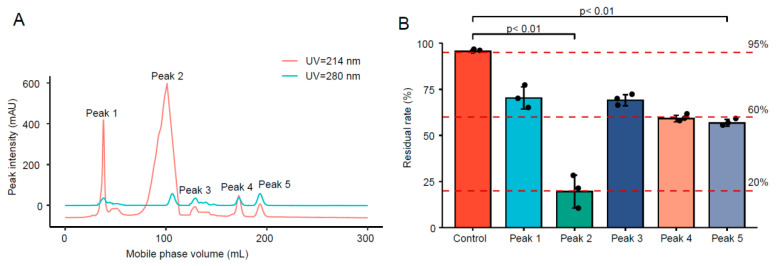
Protein separation, purification, and degradation activity of crude enzyme solution. (**A**) *ÄKTA* separation and purification results; (**B**) screening of protein degradation ability after purification.

The results are shown in Figure 1A. The *ÄKTA* protein purification and separation system was used to separate and purify the extracellular enzyme solution of ZEN-S-FS10, whose Sequencing and splicing results were in Table S2. At UV = 214 nm and UV = 280 nm, five peaks were observed and collected at 1.5 CV, suggesting five potential proteins. We noticed that Peak 2 formed a single and distinct peak shape at UV = 280 nm and a relatively broad peak shape under UV = 214 nm, suggesting a high content of amino acids and peptide bonds that would be consistent with an enzyme. The cystine formed by oxidation of Trp, Lys, and Cys residues in a protein has light absorption at UV = 280 nm; this signal can be used for protein detection or quantification [20,21]. However, proteins that do not contain Trp, Lys, or disulfide bonds have no light absorption at UV = 280 nm; thus, UV = 214 nm detection may be a better alternative [22].

#### 3.1.2. Protein Concentration and Enzyme Activity after Purification

The concentration and enzyme activity after purification were determined for the different fractions. Previous studies have shown that protein isolation using the *ÄKTA* separation has high-recovery rates [23].The results are shown in Table 1. Of the five peaks, Peak 1 showed the highest protein concentration, but Peak 2 had the highest enzyme activity.

#### 3.1.3. Degradability of Protein after Separation and Purification

The separated protein fractions were tested for the ability to degrade ZEN, and the residual content of ZEN was detected by HPLC to calculate the degradation effect. The results are shown in Figure 1B. The degradation effect of the Peak 2 sample was significantly better than other samples, suggesting that the protein in this fraction is the main protein degrading ZEN.

### 3.2. SDS-PAGE Analysis

**Figure 2:**
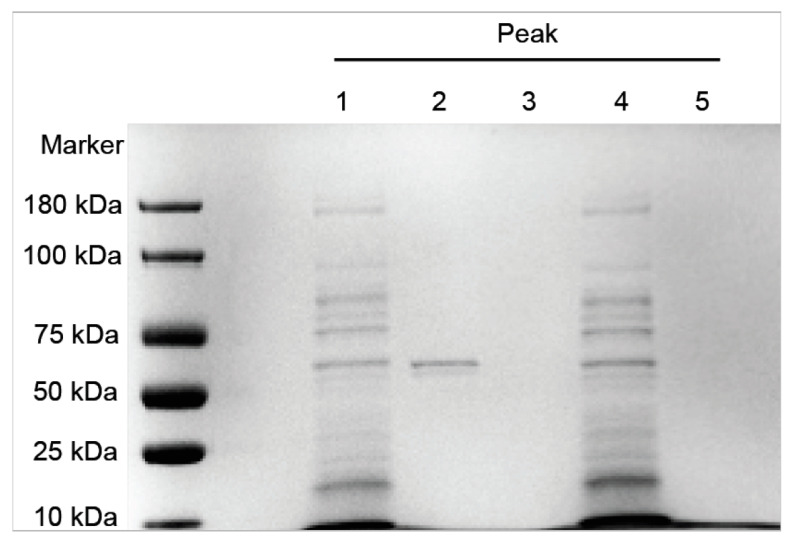
SDS-PAGE of the isolated fractions.

The five protein fractions were separated by SDS-PAGE, and the results are shown in Figure 2. Protein bands were obvious for Peak 1 and Peak 2; however, there were no obvious bands for Peak 3-Peak 5. Because the Peak 2 fraction showed the highest activity, we concluded that this fraction contains the target protein, FSZ, with molecular weight of 62.4 kDa.

### 3.3. Determination of the Optimal Active Conditions for FSZ

**Figure 3:**
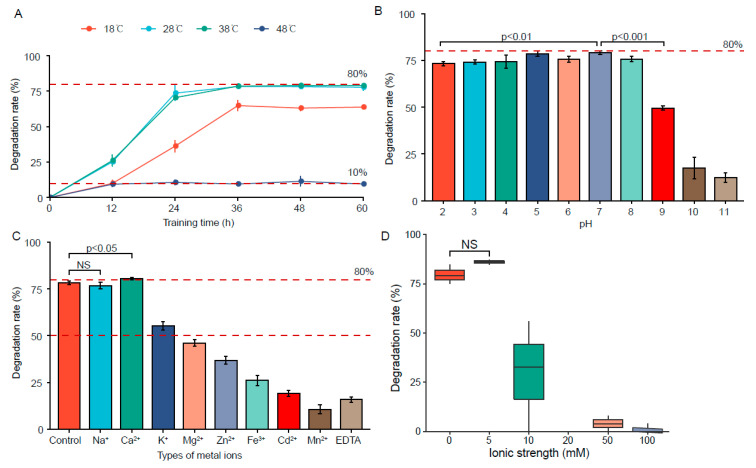
Optimal degradation conditions of FSZ: (**A**) temperature; (**B**) pH; (**C**) metal ions; (**D**) ionic strength.

The ability of FSZ to degrade ZEN under different conditions was analyzed. Temperature, pH, metal ion influence, and ionic strength were varied. The results are shown in Figure 3.

#### 3.3.1. Effect of Temperature

The effect of temperature on FSZ activity was determined, as shown in Figure 3A. The reactions were incubated at 18 °C, 28 °C, 38 °C, and 48 °C. The residual amount of ZEN was detected every 12 h, and the degradation rate was calculated. At 18 °C, the degradation rate increased with the increase of degradation time and then gradually stabilized. The same patterns were observed for degradation at 28 and 38 °C, but higher maximal rates were obtained, suggesting the optimal degradation temperature is 28–38 °C (human body temperature is 37 °C). At 48 °C, the degradation rate was greatly reduced, suggesting that high temperature reduces the enzymatic activity of FSZ to degrade ZEN. This indicates that FSZ is a heat-labile enzyme.

#### 3.3.2. Effect of pH

The effects of pH on the ability of isolated FSZ to degrade ZEN were next determined by preparing reactions with pH = 2.0–11.0. After incubation for 28 h, degradation was assayed, and the results are shown in Figure 3B. At pH = 2, the degradation rate decreased only slightly, indicating that FSZ can still maintain a strong ZEN degradation effect under acidic conditions. Therefore, a good degradation effect could be maintained in human gastric juice. At pH = 7, the degradation rate reached the maximum value. However, at higher pH (>8), the degradation rate was significantly reduced. Thus, we infer that FSZ cannot tolerate an alkaline environment and is an acid-resistant, ZEN-degrading enzyme.

#### 3.3.3. Effect of Metal Ions

The effect of different metal ions was tested, and the results are shown in Figure 3C. Compared with the blank group (control), the presence of Na^+^ had little effect on the degradation ability of FSZ. Ca^2+^ significantly promoted the degradation ability of FSZ to ZEN. Other metal ions exhibited inhibitory effects on the degradation ability of FSZ. This is consistent with the reported properties of the ZEN-degrading enzymes, POD [6] and ZENG [2] in the presence of Na^+^. Therefore, when FSZ is applied in an actual production process, the removal of metal ions may be required to promote the degradation process of FSZ.

#### 3.3.4. Effect of Ionic Strength

The effect of ionic strength on ZEN degradation by FSZ was tested, and the results are shown in Figure 3D. The 5 mM group exhibited a somewhat better rate of ZEN degradation by FSZ compared with the control group, though this effect was not significant. At ionic strength of 10 mM, ZEN degradation rate showed a significant decline. With the increase of ionic strength, the degradation rate of ZEN was generally low and approached 0%. The results suggest that FSZ cannot be applied for ZEN degradation in a high-ionic-strength environment.

### 3.4. Degradation Kinetic Analysis and Parameter Determination

**Figure 4:**
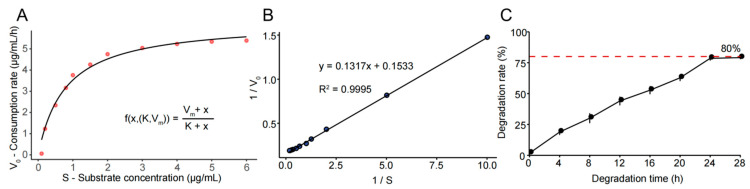
Degradation kinetics of FSZ: (**A**) nonlinear fitting of FSZ’s Michaelis equation; (**B**) Lineweaver–Burk (LB plot) of FSZ degradation; (**C**) dynamic degradation kinetic curve of FSZ degradation.

The K_m_ and V_max_ values were determined from the degradation data of FSZ at 6.0 U/mg, as shown in the double reciprocal LB diagram in Figure 4A. The ZEN degradation rate was calculated under different substrate concentrations, and nonlinear fitting was performed using Rstudio 3.6.2 software. The result satisfies the Michaelis equation. As shown in Figure 4B, linear fitting was performed on $\frac{1}{v_{0}}$ and $\frac{1}{S}$ to calculate V_max_ = 6.52 μg·mL^−1^·h^−1^ and K_m_ = 0.85 μg/mL. These values were compared with those of other enzymes previously reported to be able to degrade ZEN. The V_max_ of FSZ is 3.72 times higher than that of POD, and the K_m_ of FSZ is 2.5 times higher than that of POD [6]. Compared with other ZEN-degrading enzymes [24], FSZ is 8.25 times higher than them. Therefore, FSZ is a degrading enzyme that can efficiently degrade ZEN. As shown in Table 2, we compared the degradation properties of FSZ with other known ZEN-degrading enzymes, and the results showed that both the degradation rate and kinetic parameters of FSZ were superior to known ZEN-degrading enzymes.

Figure 4C shows the degradation rate of FSZ under the highest enzyme activity (6.0 U/mg) from 0 h to 28 h. The results show that the degradation rate of FSZ to ZEN can be stabilized at 24 h at about 80%, and the degradation rate of the first 24 h continues to increase. This indicates that the optimal degradation treatment time of FSZ is 24 h.

### 3.5. Degradability of FSZ to ZEN Derivatives

**Figure 5:**
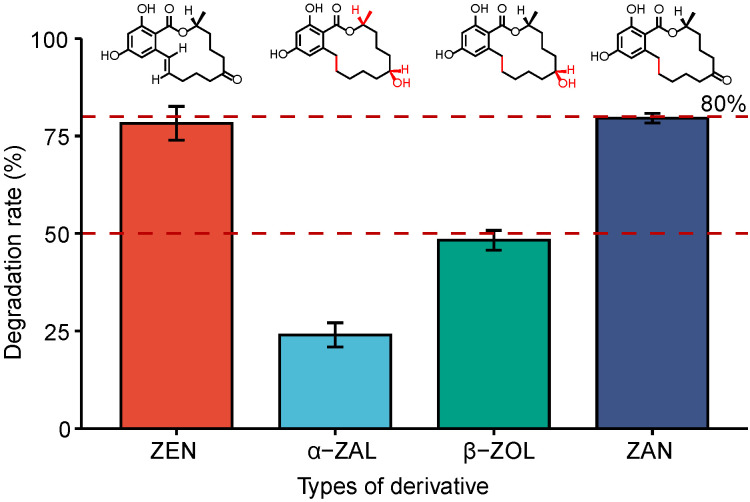
FSZ degradation of ZEN derivatives.

During actual production, changes in food production and processing conditions can cause ZEN to undergo different oxidation reactions leading to many ZEN derivatives. Therefore, effective enzymes should be able to target ZEN as well as its derivatives, such as α-ZAL and β-ZOL. The abilities of FSZ to degrade ZEN derivatives were tested, as shown in Figure 5. The ability of FSZ to degrade α-ZAL was weak, but FSZ exhibited a degradation effect of about 50% for β-ZAL and a higher degradation rate for ZAN. Therefore, the results show that FSZ can effectively degrade ZEN derivatives, allowing it to be more effective under actual food production conditions.

### 3.6. Analysis of Products after FSZ Degradation of ZEN

**Figure 6:**
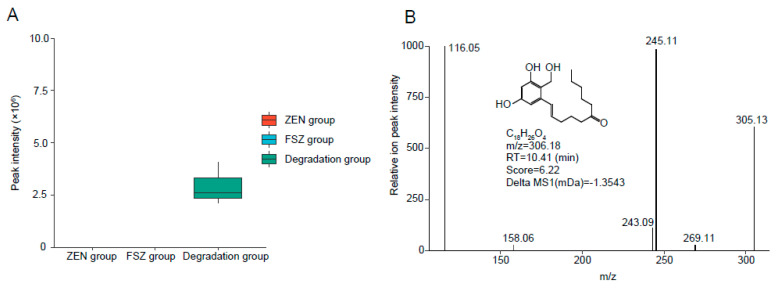
Exploration of ZEN degradation products: (**A**) analysis of the content change of the degradation product FSZ−P; (**B**) structural information analysis of the degradation product FSZ−P.

The degradation products were analyzed by UPLC-qTOF-MS, and the results are shown in Figure 6. One possible degradation product was isolated, and it was named as FSZ−P (C_18_H_26_O_4_). FSZ−P was monitored in negative-ion mode. The results are shown in Figure 6A; FSZ−P could not be detected by the instrument in the ZEN standard group (ZEN group); thus, it can be shown that this substance does not exist in the pure ZEN product. Additionally, the substance could not be detected in the FSZ group, which indicated that the product was not derived from the FSZ sample. However, this product could be detected in the degradation group after the degradation of ZEN by FSZ, which indicated that FSZ−P was produced by the degradation of ZEN by FSZ.

In addition, as shown in Figure 6B, we used MSDIAL 4.24 to analyze the main fragment ion peaks of product A and inferred its structure by MSFINDER 3.46. After uploading the data to the NCBI website, we learned that the molecular weight of this product is 306.18 g/mol, which is due to ZEN. The cleavage of the O atom in the epoxy lactone ring of the molecule results in the formation of a ring-opening product. However, this product has not been reported in the degradation process of ZEN; thus, it may be a new degradation product. In addition, since this degradation product was still present at higher levels in the samples of the FSZ group and due to the disruption of the oxygen-containing lactone ring, the estrogenic toxicity of ZEN has been lost [26,27]. Therefore, this product may be the product after the action of a single FSZ; thus, it can be the focus of this study. However, the structural information of the product still needs to be confirmed by nuclear magnetic resonance and other methods.

### 3.7. Sequence Homology Comparison and Structural Analysis of FSZ

**Figure 7:**
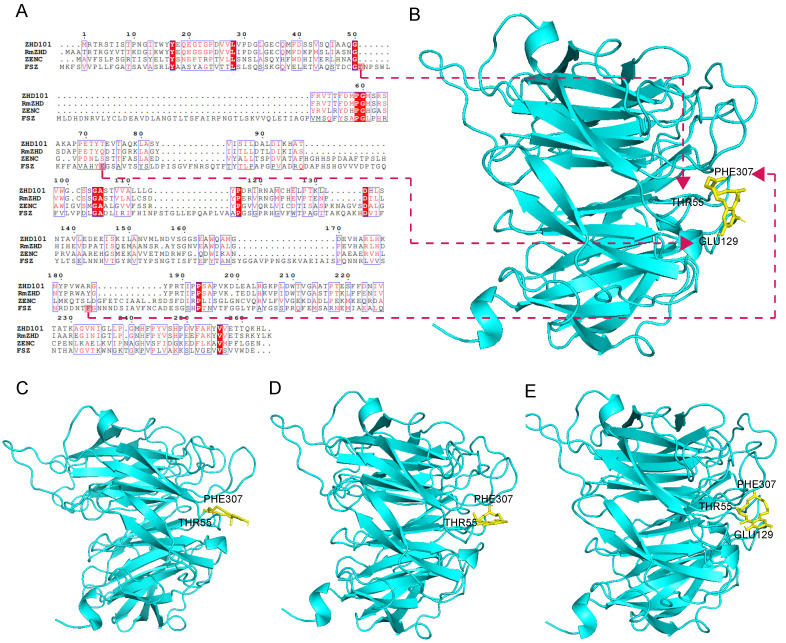
FSZ amino acid sequence alignment results and molecular docking of ZEN and derivatives: (**A**) amino acid sequence alignment of FSZ with other ZEN-degrading enzymes, ZHD101, RmZHD, and ZENC and FSZ amino acid sequence alignment; (**B**) molecular-docking results of FSZ and ZEN; (**C**) molecular-docking results of FSZ and α-ZAL; (**D**) molecular-docking results of FSZ and β-ZOL; (**E**) molecular-docking results of FSZ and ZAN.

The amino acid sequence of FSZ and other data are shown in Table S3. The detected amino acid sequence was uploaded to the NCBI website. Blast analysis indicated that FSZ, derived from *Aspergillus niger*, is a novel protein. The protein sequence of FSZ is shown in Table S4. The Clustal algorithm was used to compare the amino acid sequences of ZEN-degrading enzymes, ZHD101 [5], ZENC [9], and RmZHD [7], as shown in Figure 7A; a red-filled box indicates the same amino acid composition and a blue box indicates that the amino acid composition is partially identical. The amino acid sequence of FSZ was only 10% similar to the sequences of the other three ZEN-degrading enzymes, indicating this is a novel ZEN-degrading enzyme.

Structural simulation of FSZ by SWISS-Model (Figure S1), and the molecular docking was performed (Figure 7B). The results suggest that FSZ has three binding sites for ZEN molecules: PHE307, THR55, and GLU129. These three residues can help FSZ bind to ZEN. We also docked FSZ with derivatives of ZEN (α-ZAL, β-ZOL, and ZAN), and the results are shown in Figure 7C-7E. The docking indicated that α-ZAL and β-ZOL can only interact with PHE307 and THR55 and not to the GLU129 site. However, ZAN can interact with the above three sites (PHE307, THR55, and GLU129). Therefore, α-ZAL and β-ZOL may be unable to interact with GLU129, explaining the observed low degradation rate (<50%) of these two derivatives by FSZ. However, ZAN can bind to all three sites at the same time; thus, there is no significant difference in the rates of degradation for ZAN and ZEN. This difference in binding may be because the structural end groups of α-ZAL and β-ZOL are hydroxyl groups, but the structural end groups of ZEN and ZAN are carbonyl groups. Previous studies have shown that the presence of hydroxyl radicals may affect the biological activity of enzymes [28,29,30]. The hydroxyl structure may affect the ability of FSZ to bind and degrade α-ZAL and β-ZOL.

### 3.8. The Ability of FSZ to Degrade ZEN in Corn Flour

FSZ was tested using corn meal containing ZEN, and the results are shown in Table 3. After 28h of incubation with corn meal containing 1.0 μg/mL ZEN, the degradation rate was 78.43%. Thus, FSZ exhibited good degradation effect in the actual sample of corn flour. Additionally, this detection method exhibited good recovery rate and high accuracy.

## 4. Conclusions

We purified FSZ, an extracellular enzyme from *Aspergillus niger* ZEN-S-FS10 that can degrade ZEN efficiently. The optimal degradation conditions of FSZ were determined as degradation temperature of 28–38 ℃ and pH ≤ 7 with effects of the presence of some metal ions. FSZ also can degrade ZEN derivatives (α-ZAL, β-ZOL, ZAN) produced during the processing of ZEN; thus, this enzyme has good application prospect. We carried out kinetic modeling of the degradation process of FSZ, and the calculated values of V_max_ = 6.52 μg·mL^−1^·h^−1^ and K_m_ = 0.85 μg/mL are significantly better than those calculated for previously reported ZEN-degrading enzymes. And one ZEN degradation product was isolated: FSZ−P(C_18_H_26_O_4_) with a relative molecular weight of 306.18 g/mol, and its element composition and structural information are briefly analyzed. We compared the amino acid sequence of FSZ with those of other ZEN-degrading enzymes and found very low homology, suggesting this is a completely new degrading enzyme. Structural simulation and molecular docking of FSZ were carried out, and three potential binding sites of ZEN were analyzed: PHE307, THR55, and GLU129. According to the molecular-docking results of FSZ with derivatives of ZEN (α-ZAL, β-ZOL, ZAN), the structural ends of α-ZAL and β-ZOL cannot interact with the amino acid site of GLU129 in FSZ due to the presence of hydroxyl groups, thus explaining the low degradation rate (<50%) of these two derivatives. ZEN and ZAN can bind to the amino acid site of GLU129 due to the presence of a carbonyl group at the end; thus, the degradation rate is high (>75%). These results provide a theoretical basis for FSZ function and suggest directions for further research and application of ZEN-degrading enzymes.

## Figures and Tables

**Table 1:** Comparison of concentration and enzyme activity of the five protein fractions.

Peak	Concentration (μg/mL)	Enzyme Activity (U/mg)
1	6023.67 ± 5.09	5.82 ± 0.11
2	5887.67 ± 4.08	5.98 ± 0.05
3	5299.67 ± 6.14	2.11 ± 0.12
4	5116.63 ± 8.05	4.13 ± 0.02
5	5860.00 ± 2.59	3.12 ± 0.07

**Table 2:** Comparison of the degradation properties of FSZ with other known ZEN-degrading enzymes.

ZEN-Degrading Enzymes	Degradation Rate (%) (24 h)	V_max_ (μg·mL^−1^·h^−1^)	K_m_ (μg/mL)	Source
FSZ	75–80	6.52	0.85	*Aspergillus niger*
POD [6]	64.9	2.39	0.56	-
Peroxidase [24]	69.4	1.9	0.16	Soybean bran
ZHD101 [25]	50	-	-	*Clonostachys rosea*

-: Data not reported in the literature.

**Table 3:** Parameters of detection of ZEN in corn flour (*n* = 3).

Analytical Parameters	ZEN
Curve equation	y = 0.0052x + 0.0062
R^2^	0.9997
LD_m_ (μg/mL)	0.05
LQ_m_ (μg/mL)	0.1
Recovery rate (%)	98.75 ± 1.11
Degradation rate in corn flour (%)	78.43 ± 1.78

LD_m_-detection limit of the method; LQ_m_-quantification limit of the method.

## Data Availability

Data is contained within the article and supplementary materials.

## Supplementary Materials

The following supporting information can be downloaded at: https://www.mdpi.com/article/10.3390/foods11182908/s1, Figure S1: Structural simulation of FSZ by SWISS-Model; Table S1: 18s rDNA sequencing of ZEN-S-FS10; Table S2: Sequencing and splicing results of ZEN-S-FS10; Table S3: Amino acid sequencing results of FSZ; Table S4: The protein sequence of FSZ; Table S5: Abbreviation.
