# Supplementary material for: Isolation and Mechanistic Characterization of a Novel Zearalenone-Degrading Enzyme
Source: Foods. 2022 Sep 19;11(18):2908. doi: 10.3390/foods11182908 (PMC9498698; doi:10.3390/foods11182908)
Supplement: Supplementary file 1 [file foods-11-02908-s001.zip › foods-1857884-supplementary.pdf]

Isolation and mechanistic characterization of a novel zearalenone-degrading enzyme

Jian Ji[ab], Jian Yu[a], Wei Xu[a], Yi Zheng[c], Yinzhi Zhang[a], Xiulan Sun[a]*

[a]School of Food Science and Technology of Jiangnan University, State Key Laboratory of Food Science and Technology, Wuxi, Jiangsu 214122, China.
[b]College of Food Science and Pharmacy, Xinjiang Agricultural University, Ürümqi, 830052 Xinjiang Uygur Autonomous Region People's Republic of China.
[c]Jiangsu Agri-animal Husbandry Vocational College, Key Laboratory for High-Tech Research and Development of Veterinary Biopharmaceuticals, Jiangsu 225300, China

First author: Jian Ji (E-mail:jijian@jiangnan.edu.cn)
Jian Yu (E-mail:jiangsuyzyj@163.com)
Corresponding author: Xiulan Sun
Tel: +86 510-85329015; fax: +86 85328726
E-mail address: sxlzyz@jiangnan.edu.cn

Supporting tables and figures

Table:

Table S1 18s rDNA sequencing of ZEN-S-FS10
Table S2 Sequencing and splicing results of ZEN-S-FS10
Table S3 Amino acid sequencing results of FSZ
Table S4 The protein sequence of FSZ
Table S5 Abbreviation

Figure：

Figure S1 Structural simulation of FSZ by SWISS-Model

Table:

Table S1 18s rDNA sequencing of ZEN-S-FS10

Name	Homology	Reference species
ZEN-S-FS10	696/703(99%)	Aspergillus niger

Table S2 Sequencing and splicing results of ZEN-S-FS10

Sequencing
TGCCCCCCGGAATACCAGGGGGCGCAATGTGCGTTCAAAGACTCGATGA<br>TTCACTGAATTCTGCAATTCACATTAGTTATCGCATTTCGCTGCGTTCTTC<br>ATCGATGCCGGAACCAAGAGATCCATTGTTGAAAGTTTTAACTGATTGCA<br>TTCAATCAACTCAGACTGCACGCTTTCAGACAGTGTTCGTGTTGGGGTC<br>TCCGGCGGGCACGGGCCCGGGGGGCAGAGGCGCCCCCCCGGCGGCCGA<br>CAAGCGGCGGGCCCGCCGAAGCAACAGGGTACAATAGACACGGATGGG<br>AGGTTGGGCCCAAAGGACCCGCACTCGGTAATGATCCTTCCGTTAGGGG<br>AACCTGCGGAAGGATCATTACCGAGTGCGGGTCCTTTGGGCCCAACCTC<br>CCATCCGTGTCTATTGTACCCTGTTGCTTCGGCGGGCCCGCCGCTTGTCG<br>GCCGCCGGGGGGGCGCCTCTGCCCCCCGGGCCCGTGCCCGCCGGAGAC<br>CCCAACACGAACACTGTCTGAAAGCGTGCAGTCTGAGTTGATTGAATGC<br>AATCAGTTAAAACTTTCAACAATGGATCTCTTGGTTCCGGCATCGATGAA<br>GAACGCAGCGAAATGCGATAACTAATGTGAATTGCAGAATTCAGTGAAT<br>CATCGAGTCTTTGAACGCACATTGCGCCCCCTGGTATTCCGGGGGGCATG<br>CCTGTCCGAGCGTCATTGCTGCCCTCAAGCCCGGCTTGTGTGTTGGGTC<br>GCCGTCCCCCTCTCCGGGGGGACGGGCCCGAAAGGCAGCGGCGGCACC<br>GCGTCCGATCCTCGAGCGTATGGGGCTTTGTCACATGCTCTGTAGGATTG<br>GCCGGCGCCTGCCGACGTTTTCCAACCATTCTTTCCAGGTTGACCTCGG<br>ATCAGGTAGGGATACCCGCTGAACTTAAGCATATCAAAAGGCCGGAGGA<br>AACCATCATAAACCTT

Table S3 Amino acid sequencing results of FSZ

Protein name	Accession	Molecular weight（kDa）	Protein score	Source	characteristic peptide sequence
FSZ（unnamed protein）	scaffold2.t77	62.4	53.5	Aspergillus niger	[R].HGVFWTPAGTTAK.[Q]

Accession：The registration number of the protein in NCBI

Characteristic peptide sequence：Characteristic encoding of this protein different from other proteins

Table S4 The protein sequence of FSZ

Sequencing
MKFSVVPLLFGATSAVASRLYAASYAGTVTTLSLSQSSKGQYELETVAQSTDCGTNPSWL<br>MLDHDNRVLYCLDEAVDLANGTLTSFAIRPNGTLSKVVQLETIAGPVMSQFYSAPGLPHR<br>KFFAVAHYEGSAVTSYSLDPISGVFNRSQTFTYTLPAPGPVADRQDAPHSHGVVVDPTGQ<br>FVLVPDLGADLIRIFHINPSTGLLEPQAPLVAAPGSGPRHGVFWTPAGTTAKQAKHDVIF<br>YLTSELNNHVTGYRVTYPSNGTISFTEFYTANSYGGAVPPNGSKVAEIAISPQNNRLVVS<br>NRDDNTFGNNNDSIAVFNCADESGSHPTNVTFGGLYPAYGSSPRQFEMSARNEMIAEALQ<br>NTHAVGVTKWNGKTGKPVPLVAKKSLVGEVVSVVWDE

Table S5 Abbreviation

Abbreviation	Full name
ZEN	Zearalenone
α-ZAL	α-Zearalanol
β-ZOL	β-Zearalenol
ZAN	Zearalanone
ZEN-S-FS10	The name of the original strain of the degrading enzyme
FSZ	Degrading enzyme name
CV	Column volume

Figure：

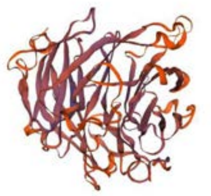

Figure S1 Structural simulation of FSZ by SWISS-Model
